# Effects of tea, catechins and catechin derivatives on Omicron subvariants of SARS-CoV-2

**DOI:** 10.1038/s41598-023-43563-3

**Published:** 2023-10-03

**Authors:** Masaharu Shin-Ya, Maiko Nakashio, Eriko Ohgitani, Akiko Suganami, Masaya Kawamoto, Masaki Ichitani, Makoto Kobayashi, Takanobu Takihara, Tohru Inaba, Yoko Nukui, Hitoshi Kinugasa, Hiroyasu Ishikura, Yutaka Tamura, Osam Mazda

**Affiliations:** 1 Department of Immunology, Kyoto Prefectural University of Medicine, Kyoto, Japan; 2 Department of Molecular Anti-Virus Immunology, Kyoto Prefectural University of Medicine, Kyoto, Japan; 3 Department of Emergency and Critical Care Medicine, Faculty of Medicine, Fukuoka University, Fukuoka, Japan; 4 Department of Bioinformatics, Graduate School of Medicine, Chiba University, Chiba, Japan; 5 Central Research Institute, ITO EN, Ltd, Shizuoka, Japan; 6 Department of Infection Control and Laboratory Medicine, Kyoto Prefectural University of Medicine, Kyoto, Japan

**Keywords:** Microbiology, Diseases

## Abstract

The Omicron subvariants of SARS-CoV-2 have multiple mutations in the S-proteins and show high transmissibility. We previously reported that tea catechin (−)-epigallocatechin gallate (EGCG) and its derivatives including theaflavin-3,3’-di-O-digallate (TFDG) strongly inactivated the conventional SARS-CoV-2 by binding to the receptor binding domain (RBD) of the S-protein. Here we show that Omicron subvariants were effectively inactivated by green tea, *Matcha,* and black tea. EGCG and TFDG strongly suppressed infectivity of BA.1 and XE subvariants, while effect on BA.2.75 was weaker. Neutralization assay showed that EGCG and TFDG inhibited interaction between BA.1 RBD and ACE2. In silico analyses suggested that N460K, G446S and F490S mutations in RBDs crucially influenced the binding of EGCG/TFDG to the RBDs. Healthy volunteers consumed a candy containing green tea or black tea, and saliva collected from them immediately after the candy consumption significantly decreased BA.1 virus infectivity in vitro. These results indicate specific amino acid substitutions in RBDs that crucially influence the binding of EGCG/TFDG to the RBDs and different susceptibility of each Omicron subvariant to EGCG/TFDG. The study may suggest molecular basis for potential usefulness of these compounds in suppression of mutant viruses that could emerge in the future and cause next pandemic.

## Introduction

The B.1.1.529 lineage of the severe acute respiratory syndrome coronavirus 2 (SARS-CoV-2) was identified in South Africa in November 2021, and designated as the Omicron variant by the World Health Organization (WHO)^1,2^. In comparison with the original strain, the Omicron BA.1 variant harbored 30 amino acid substitutions, deletion of six amino acid residues and an insertion of three amino acid residues in the spike protein that plays key roles in the viral attachment and entry into cells^1–4^. Due to these heavy mutations, the Omicron variant was recognized as a variant of concern (VOC) with high transmissibility and potential to cause breakthrough infection in vaccinated individuals^3–5^. Indeed, infection of this variant spread worldwide in 2022, replacing previous variants of SARS-CoV-2. Thereafter, sublineages of the Omicron variant including BA.2, BA.5, BA.2.75 and XBB.1 evolved with additional mutations and caused widespread infection (Supplementary Fig. S1).

SARS-CoV-2 is mainly transmitted by saliva of COVID-19 patients and asymptomatic infected persons, because SARS-CoV-2 infects, and propagates in, salivary glands and oral mucosa in human^6,7^. Saliva containing the virus may be scattered by speaking, sneezing and coughing and forms droplets and aerosols that could reach nasal and oral mucosa of nearby persons who may subsequently get infected^8–15^. We considered that inactivation of virus in saliva should be important, and explored various foods and food ingredients that inactivate the conventional strain of SARS-CoV-2^16^. We reported that exposure of the virus to green tea, roasted green tea, oolong tea and black tea in vitro resulted in significant reduction of the virus infectivity^16–18^. We also found that the tea catechin compound (−)- epigallocatechin gallate (EGCG) powerfully and rapidly inactivated the virus^16–18^, which was also reported by other groups^19–21^. Similar effects were also seen in black tea ingredients, galloylated theaflavins (theaflavin-3-O-gallate (TF3G), theaflavin-3’-O-gallate (TF3’G), and theaflavin-3,3’-O-digallate (TFDG)), and theasinensin A (TSA) that are derivatives of tea catechins^17,22^. We also reported that the EGCG, TFDG and TSA interfered with the interaction between viral spike protein and ACE2 by binding to the spike protein receptor binding domain (RBD). However, it remains to be clarified whether these tea ingredients also inactivate the Omicron variants of SARS-CoV-2. Moreover, it has not been elucidated which amino acid substitutions in the Omicron subvariants may influence the binding of EGCG and TFDG to the spike protein.

In this study we examined the efficiencies of tea, tea catechins and catechin derivatives to inactivate the Omicron subvariant strains of the SARS-CoV-2. We also assessed whether saliva from healthy volunteers who consumed a candy containing green tea or black tea inactivates BA.1 in vitro.

## Results

### Decrease in infectivity of omicron subvariants by treatment with tea

**Figure 1:**
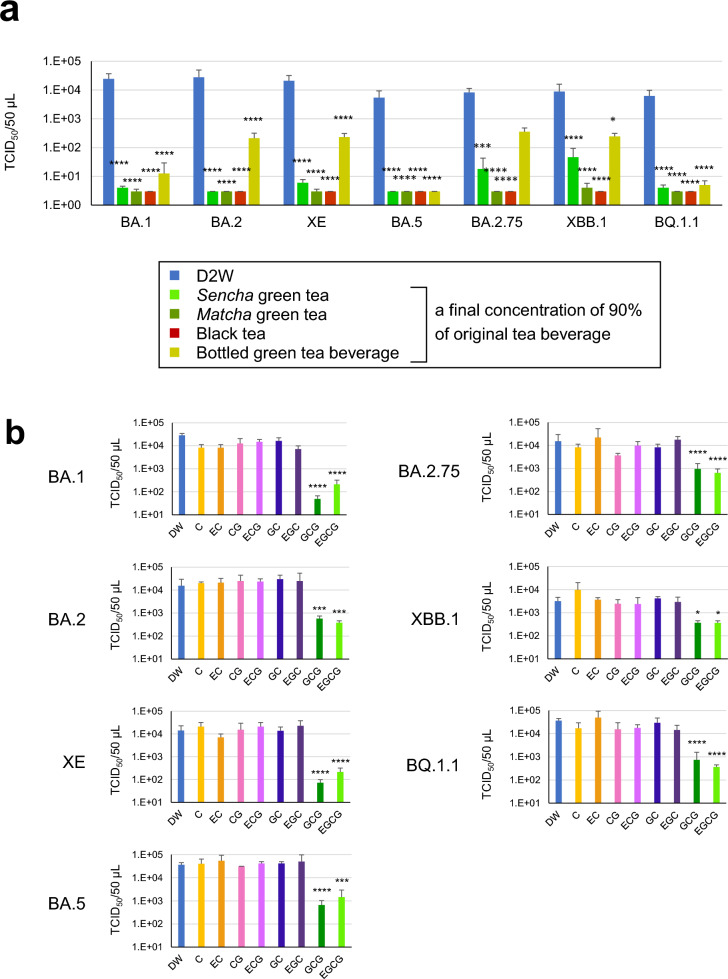
Analysis of inactivation effects of tea and green tea catechins on SARS-CoV-2 Omicron variants. Viruses were treated with the indicated tea (**a**) or each catechin at the same concentration in bottled green tea beverage (Supplementary Table 1) (**b**) for 10 s. After dilution, the virus was infected to VeroE6/TMPRSS2 cells to determine TCID_50_ values as described in the Materials and Methods. Virus titer of each sample (means ± S.D.) is shown (n = 4). ****p < 0.0001, ***p < 0.001, **p < 0.01, and *p < 0.05 vs. Control (distilled water) (Tukey’s multiple comparisons test).

To estimate whether Omicron subvariants of SARS-CoV-2 were inactivated by tea, the virus suspension was exposed to green tea or black tea that was freshly brewed by pouring hot or boiled water over tea leaves, or to *Matcha* green tea that was freshly prepared by whisking powdered *Matcha* tea in hot water, just like preparation of ordinary beverage (see “Materials and Methods”). The virus suspension was mixed with the original tea beverage at the final concentration of × 9/10 (90%). After 10 s, the tea/virus mixtures were serially diluted and subjected to TCID_50_ assay to determine infectivity of the virus. As shown in Fig. 1a, the titer of BA.1 dropped to less than 1/100 of that of DW-treated control virus by the treatment with any of these tea samples. Similar results were also seen using other subvariants.

Meanwhile, a bottled green tea beverage remarkably decreased infectivity of BA.1, BA.5 and BQ.1.1 but failed to do so on BA.2.75 subvariant. Virus titers of BA.2 and XE were reduced to approximately 1/100 after treatment with a bottled green tea beverage.

These results were not caused by artificial effects due to different concentrations of fresh culture medium in the wells nor by toxic effects of tea on the VeroE6/TMPRSS2 cells (Supplementary Fig. S2).

### EGCG and GCG effectively inactivated some but not all omicron subvariants

We treated Omicron subvariants with various tea catechins for 10 s, and evaluated TCID_50_ values. Catechins were contained in green tea at different concentrations. Virus was treated with each catechin at the same concentration in the bottled green tea beverage that we used (Supplementary Table S1). For an example, to treat virus with EGCG, the concentration of EGCG was adjusted to 641 µM that is the same as the EGCG concentration in the bottled green tea beverage. ( +)-catechin (C), ( −)-epicatechin (EC), ( −)-catechin gallate (CG), ( −)-epicatechin gallate (ECG), ( −)-gallocatechin (GC), and ( −)-epigallocatechin (EGC) did not significantly affect the infectivity of all the Omicron subvariants under these experimental conditions (Fig. 1b). In contrast, EGCG and its epimer, ( −)-gallocatechin gallate (GCG), reduced the virus titers of BA.1 and XE to less than 1/100, and declined the infectivity of BA.2 and BA.2.75 to approximately 1/10. These anti-virus effects of EGCG were comparable to, or weaker than, those of the bottled green tea beverage (Fig. 1a and b).

**Figure 2:**
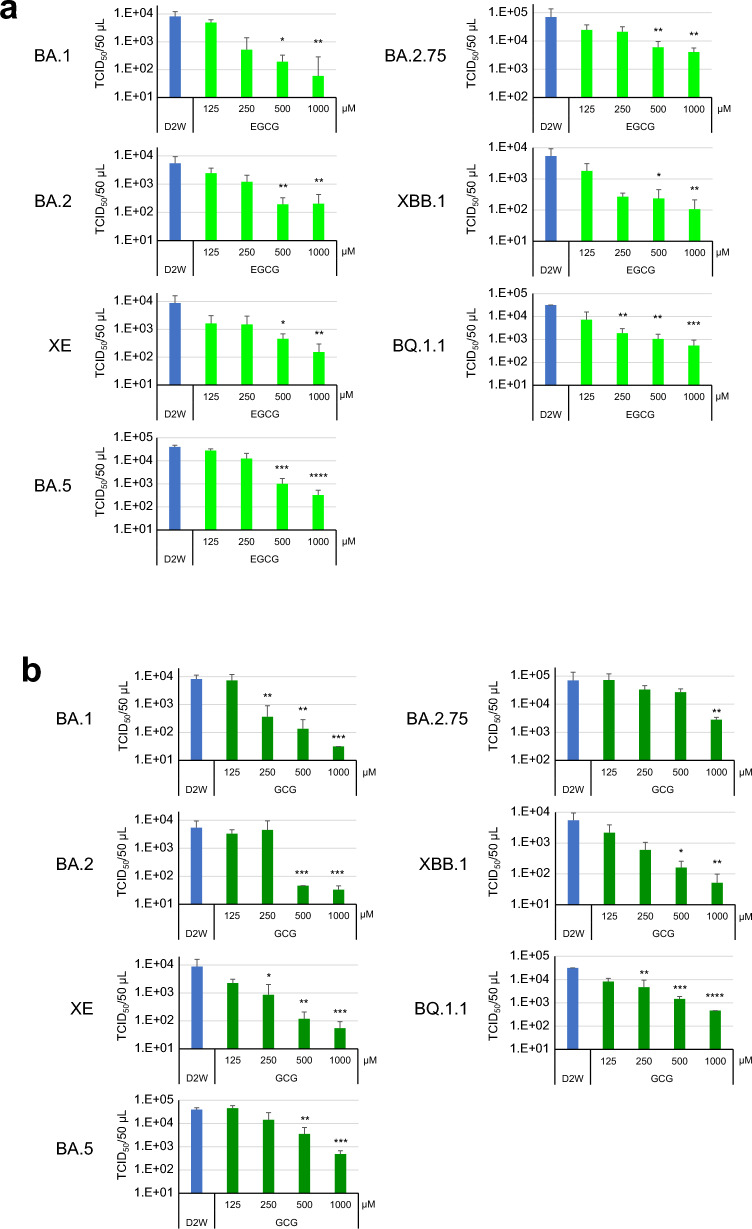
Effect of EGCG and GCG on Omicron subvariants. Omicron subvariants were treated with the indicated concentrations of EGCG (**a**) or GCG (**b**) for 10 s. After dilution, virus titers (means ± S.D.) were determined as in Fig. 1 (n = 4). ****p < 0.0001, ***p < 0.001, **p < 0.01, and *p < 0.05 vs. Ctrl (distilled water) (Tukey's multiple comparisons test).

We also examined the effect of various concentrations of EGCG and GCG on the Omicron subvariants. As shown in Fig. 2a, EGCG at 1000 µM inactivated more than 99% of BA.1 and BA.5 viruses, whereas more than 1% of BA.2, BA.2.75, XBB.1 and BQ.1.1 viruses remained infectious after the same treatment. Similarly, GCG at 1000 µM decreased virus titers of BA.1, XE and XBB.1 to less than 1/100, while its effects on BA.5, BA.2.75 and BQ.1.1 were less potent with the reduction rates between 1/10 and 1/100 (Fig. 2b).

### Effects of theaflavins on infectivity of omicron subvariants

**Figure 3:**
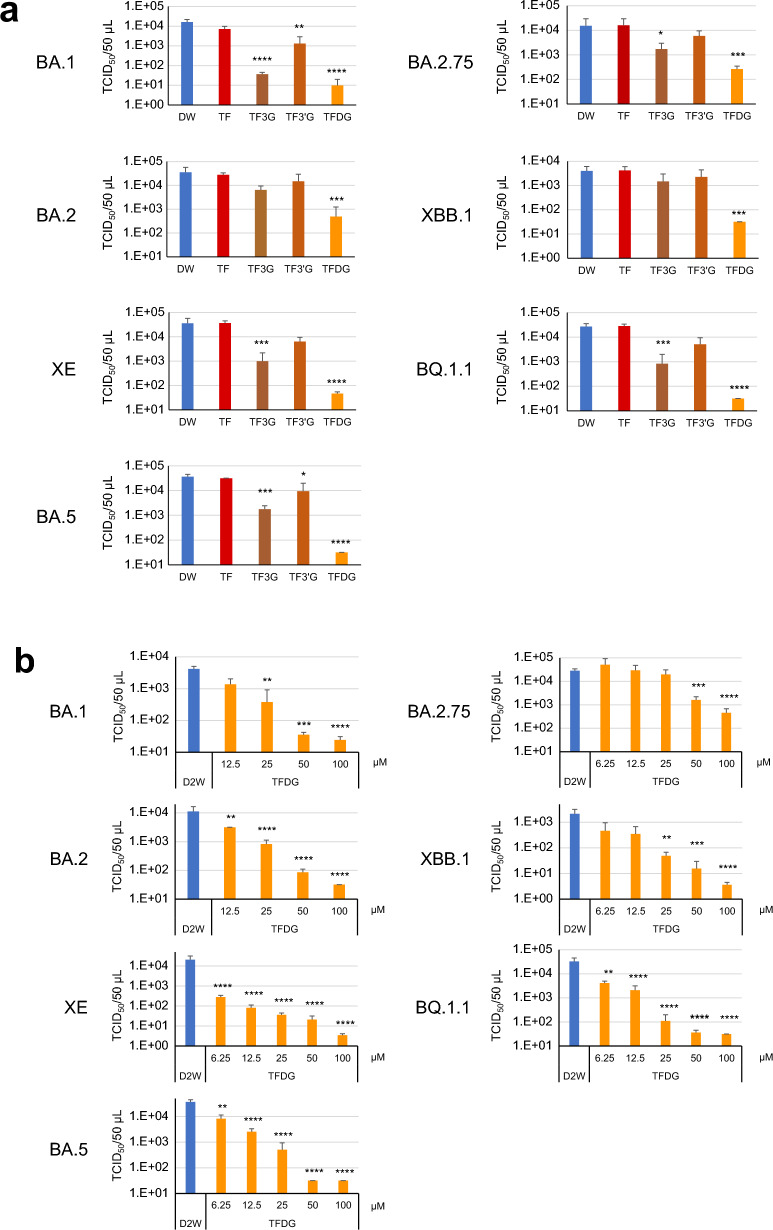
Effect of theaflavins on Omicron subvariants. Viruses were treated with each compound at the same concentration in black tea (**a**) or with the indicated concentrations of TFDG (**b**) for 10 s. After dilution, virus titers (means ± S.D.) were determined as in Fig. 1 (n = 4). ****p < 0.0001, ***p < 0.001, **p < 0.01, and *p < 0.05 vs. Ctrl (distilled water) (Tukey’s multiple comparisons test).

Omicron subvariants were treated with theaflavins (TF, TF3G, TF3’G, TFDG) at the concentrations equivalent to those in typical black tea (Supplementary Table S1)^17^. TCID_50_ assay revealed that TFDG remarkably decreased the virus titers of BA.1, XE, BA.5, XBB.1 and BQ.1.1, whereas the reduction rates of BA.2 and BA.2.75 were less significant (between 1/10 and 1/100) (Fig. 3a). TF3G reduced infectivity of BA.1 to < 1/100 without affecting other Omicron variants at similar degrees. Neither non-galloylated TF nor TF3’G reduced virus titers to less than 1/100.

We also examined inactivation effects of various concentrations of TFDG on the Omicron subvariants. As shown in Fig. 3b, TFDG at 50 or 100 µM reduced the infectivity of most of the subvariants to 1/100 or less, except that more than 1% of the BA.2.75 was not inactivated by the treatment with 100 µM of the compound.

### EGCG inactivated virus but did not render cells resistant to the virus

**Figure 4:**
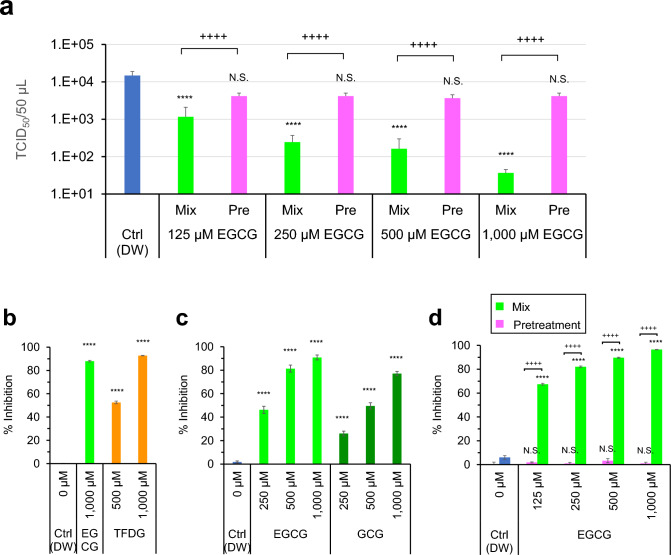
EGCG, GCG, and TFDG hampered the interaction between ACE2 and RBD of Omicron BA.1. (**a**) BA.1 virus and EGCG was mixed, and the mixture was infected to cells (“Mix”), while other cells were pre-treated with EGCG followed by washing and infection by DW-treated BA.1 virus (“Pre”). As a control (DW), DW-treated BA.1 virus was infected to non-treated cells. Virus titers were determined as in Fig. 1. (**b**–**d**) HRP-RBD was mixed with EGCG, and the mixture was added to ACE2-pre-coated wells (“Mix protocol”) (**b**,**c**), and green bars in (**d**), while other ACE2-pre-coated wells were pretreated with EGCG followed by washing and an addition of DW-treated HRP-RBD (“Pretreatment protocol”) (pink bars in (**d**)). As a control (DW), DW-treated HRP-RBD was added to ACE2-pre-coated wells. The binding between RBD and ACE2 was evaluated, and % Inhibition for each sample is shown. Values are means ± S.D. (n = 3). ****p < 0.0001 vs. Control (DW), N.S., p > 0.05 vs. Control (DW), +  +  +  +p < 0.0001, between indicated groups, by Tukey’s multiple comparison test.

We examined the possibility that anti-virus effect of EGCG was seen because EGCG acted on cells rather than on virus. To clarify this point, we pretreated the cells with EGCG, before washing and infection with BA.1 virus that had been treated with distilled water (DW) (“Pre” protocol). As shown in the Fig. 4a, virus titer was not significantly reduced. The result was in sharp contrast to the remarkable inactivation of virus that had been mixed with EGCG (“Mix” protocol in Fig. 4a that is the same procedure as performed in Figs. 1b and 2a). Therefore, it is strongly suggested that EGCG inactivated the virus but did not induce anti-virus effects in the cells.

### EGCG, GCG and TFDG inhibited interaction of BA.1 RBD with ACE2

Neutralizing assay was performed to test whether EGCG, GCG and TFDG prevented interaction between RBD of the BA.1 Spike protein and ACE2. As results, the interaction between RBD and ACE2 was strongly suppressed by EGCG, GCG and TFDG that were mixed with RBD followed by an addition of the resultant RBD/compound mixture to ACE2-pre-coated wells (“mix protocol”) (Fig. 4b, c, and green bars in d). In contrast, the protein interaction was not significantly inhibited if ACE2 was pretreated with EGCG before an addition of DW-treated RBD (Fig. 4d, pink bars). These data strongly suggest that EGCG inhibited interaction between RBD and ACE2 by binding to RBD rather than to ACE2.

### In silico simulation analyses

To analyze the inhibition of the physical association of Omicron RBD of S-protein with ACE2 by EGCG and TFDG, we employed in silico molecular docking simulation to elucidate how EGCG and TFDG abolished the interaction of the Omicron RBD of S-protein with ACE2^23,24^. According to Han P et al.^25^, the contact between the RBD of surface spike glycoprotein (S-protein) of SARS-CoV-2 and the extracellular peptidase domain of ACE2 can be divided into two patches, Patch 1 and Patch 2. At Patch 1, Y453, A475, N477, F486, N487, Y489, and R493 of the RBD form a network of H-bonds with S19, Q24, F28, H34, E35, L79, M82, and Y83 from ACE2. Besides, at Patch 2, Y449, R498, T500, Y501, and G502 of the RBD is H-bonded to D38, Y41, Q42, and K353 of ACE2. Han et al.^25^ also reported that K417N, G446S, E484A, G496S, and Y505H substitutions decrease the binding affinity of Omicron RBD with hACE2.

A comparative study of amino acid mutation status in the RBD domains of seven mutants revealed that all seven strains shared the K417N, E484A, and Y505H substitutions (Supplementary Fig. S1). Meanwhile, the results of in vitro analysis (Fig. 2a) suggested that the N460K substitution may be correlated with the inactivation of the virus by EGCG.

**Figure 5:**
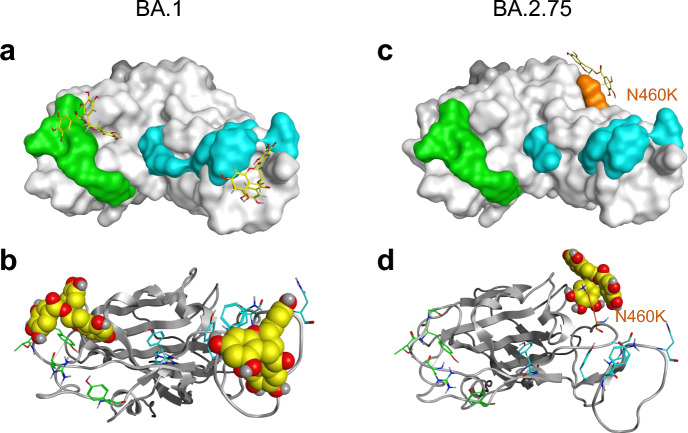
Structure of EGCG binding with the Omicron RBD of the BA.1 and BA.2.75 lineages. (**a**) EGCG (Stick model as yellow) on the binding surface of RBD of the BA.1 lineage (Surface model). (**b**) EGCG (Space-filling model) on the binding surface of RBD of the BA.1 lineage (Skelton model). (**c**) EGCG (Stick model as yellow) on the binding surface of RBD of the BA.2.75 lineage (Surface model). (**d**) EGCG (Space-filling model) on the binding surface of RBD of the BA.2.75 lineage (Skelton model). The residues in Patch 1 of RBD are shown as cyan sticks, and those in Patch 2 of RBD are shown as green sticks. The residue (N460K) mutated in the BA.2.75 lineage is shown as orange.

We, therefore, classified the seven mutants according to the N460K substitution into two groups, Group 1 (without N460K mutation; BA.1, BA.2, XE, and BA.5 lineages) and Group 2 (with N460K mutation; BA.2.75, BQ1.1, and XBB.1 lineages). BA.1 and BA.2.75 were arbitrarily chosen as representative Group 1 and Group 2 virus lineages, respectively, and ICA analyses were performed for these strains (Fig. 5).

As for the BA.1 (Group 1), EGCG could interact with either Patch 1 (any of Y453, A475, N477, F486, N487, Y489, and R493) or Patch 2 (any of Y449, R498, T500, Y501, and G502) depending on the situation (Fig. 5a,b). Both the galloyl group and 3,4,5-trihydroxyphenyl group of EGCG may be involved in the interaction between EGCG and the RBD of Spike protein of Group 1 viruses, and this may explain why Group 1 viruses were inactivated by EGCG and GCG but not by other catechins (Fig. 5a,b and Supplementary Fig. S3).

Figure 5c and d show the interaction between EGCG and RBD of BA.2.75 (Group 2). The N460K is located outside Patch 1 and Patch 2, presumably making it difficult for EGCG to interfere with the physical association between the Omicron RBD of the S protein of Group 2 and ACE2.

**Figure 6:**
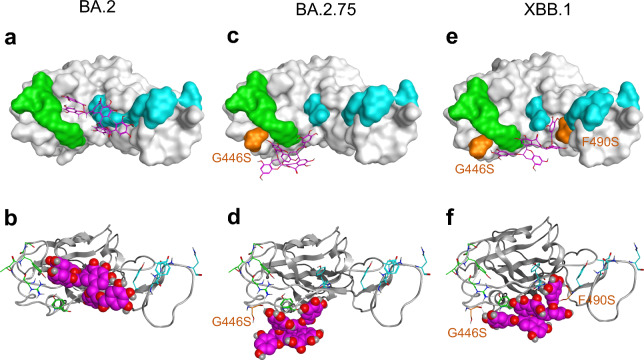
Structural comparison of TFDG binding with the Omicron RBD. (**a**) TFDG (Stick model as Magenta) on the binding surface of RBD of the BA.2 lineage (Surface model). (**b**) TFDG (Space-filling model) on the binding surface of RBD of the BA.2 lineage (Skelton model). (**c**) TFDG (Stick model as Magenta) on the binding surface of RBD of the BA.2.75 lineage (Surface model). (**d**) TFDG (Space-filling model) on the binding surface of RBD of the BA.2.75 lineage (Skelton model). (**e**) TFDG (Stick model as Magenta) on the binding surface of RBD of the XBB.1 lineage (Surface model). (**f**) TFDG (Space-filling model) on the binding surface of RBD of the XBB.1 lineage (Skelton model). The residues in Patch 1 of RBD are shown as cyan sticks, and those in Patch 2 of RBD are shown as green sticks. The G446S and F490S mutated residues are shown as orange.

The G446S substitution was associated with BA.1, BA.2.75, and XBB.1 lineages, while the G496S substitution was observed only in BA.1. Considering the in vitro analysis using TFDG (Fig. 3b), we classified the seven mutants according to the G446S substitution into two groups, Group 1’ (without G446S substitution; BA.2, XE, BA.5, and BQ1.1 lineages) and Group 2’ (with G446S substitution; BA.1, BA.2.75, and XBB.1 lineages) (Supplementary Fig. S1). BA.2 and BA.2.75 were arbitrarily chosen as representative Group 1’ and Group 2’ viruses, respectively (Fig. 6).

In BA.2, as a representative of Group 1’, it was observed that TFDG interacted with Y453, F486, and Q493R in Patch 1, Y449, Q498R, and N501Y in Patch 2 (Fig. 6a,b). The interaction of TFDG with Y449, Y453, F486, Q493R, Q498R, and N501Y of BA.2 seems to intercept a network of hydrogen bonds with H34, E35, D38, Y41, Q42, L79, M82, Y83, and K357 from ACE2.

As for BA.2.75, as typical of Group 2’, it was observed that TFDG interacted with Q493R in Patch 1, G446S, Y449, and Q498R in Patch 2 (Fig. 6c,d). The interaction of TFDG with Y449, Q493R, and Q498R of BA.2.75 seems to intercept a network of hydrogen bond with H34, E35, D38, Y41, and Q42 from ACE2.

We analyzed XBB.1 RBD separately from other Group 2’ subvariants, because it has F490 S substitution in addition to the G446S. This may allow TFDG to bind to both Patch 1 and 2 of the RBD of the XBB.1 S-protein (Fig. 6e,f).

TFDG contains two galloyl groups (at positions 3 and 3’), and both of them may be involved in the interaction of TFDG to RBDs of BA.2 (Group 1’) and XBB.1 (Fig. 6a,b,e,f and Supplementary Fig. S4). This may explain why these viruses were efficiently inactivated by TFDG but not by TF3G, TF3’G, nor TF (Fig. 3a). With regard to the interaction between TFDG and RBD of Group 2’ viruses (Fig. 6c and d), only one galloyl group of TFDG (at position 3) may play an important role (Supplementary Fig. S4), and this may explain different activities of TF3G and TF3’G to inactivate BA.1 that belongs to Group 2’ viruses (Fig. 3a).

### BA.1 virus was inactivated by saliva from volunteers who consumed a candy containing tea

**Figure 7:**
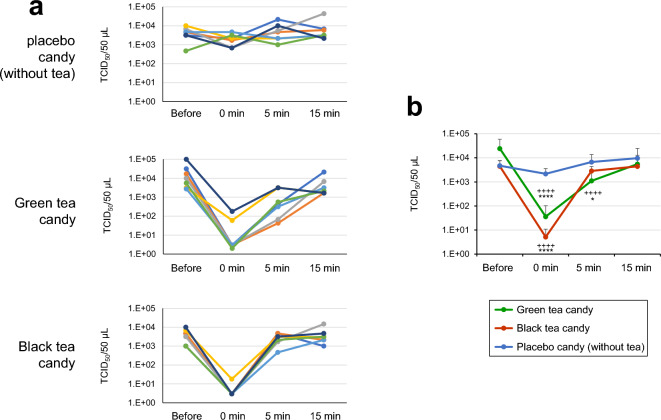
Inactivation of BA.1 Virus in vitro by Saliva from Volunteers Who Consumed a Candy Containing Tea. Healthy volunteers consumed a placebo candy (without tea) or a candy containing either green tea or black tea for 5 min. Saliva was collected from the volunteers before, or the indicated time after cessation of, the candy consumption. BA.1 virus was treated with each saliva sample for 10 s. After dilution, virus titers were determined by TCID_50_ method (n = 7 persons). (**a**) Kinetic change of virus titers for each single person is plotted. (**b**) Means ± S.D. of virus titers are shown. ****p < 0.0001, *p < 0.05, vs. placebo candy (without tea) group at the same time point. +  +  +  + p < 0.0001, vs. “before” time point of the same groups (Tukey's multiple comparisons test).

Finally, we examined whether consumption of a candy containing green tea or black tea may render the saliva capable of inactivating SARS-CoV-2. Seven healthy volunteers consumed a placebo candy (without tea) or a candy containing either green tea or black tea (the contents of the candies are shown in the Supplementary Table S4). Saliva was collected from the volunteers before the candy consumption, or after cessation of the candy consumption. BA.1 virus was treated with each saliva sample in vitro for 10 s. The virus was significantly inactivated by the treatment with saliva from healthy volunteers who consumed a candy containing green tea or black tea, provided that the saliva was collected from the volunteers immediately after the cessation of the candy consumption (Fig. 7). Five or 15 min after the cessation of the candy, the inactivation effect was almost lost in the saliva.

These results were not caused by toxic effects of sucrose on the virus or on VeroE6/TMPRSS2 cells (Supplementary Fig. S5).

High concentrations of EGCG and TFDG were demonstrated in the saliva collected from volunteers 0 min after cessation of consumption of the green tea or black tea candy, respectively (Supplementary Table S5).

## Discussion

The Omicron subvariants of SARS-CoV-2 have various mutations in the viral Spike proteins, including N501Y, E484K, E484Q, L452R^26–30^. These amino acid substitutions may be associated with the high contagiousness and/or immune escape of the variants^26,27^. We found that all the Omicron subvariants that we tested were efficiently inactivated by treatment with green tea, *Matcha* green tea, and black tea for 10 s (Fig. 1a), which is consistent with our previous study using conventional SARS-CoV-2^17,18^. A bottled green tea beverage was also effective to some subvariants, whereas its effects on other subvariants were not satisfactory, probably because concentrations of catechins and their derivatives were relatively low in this beverage compared with green tea that was freshly brewed from tea leaves by boiled water.

Each subvariant showed different sensitivity to catechins and theaflavins (Figs. 1, 2, 3). This may be due to various amino acid substitutions in the RBD of the S protein of each subvariant. The N460K substitution may crucially influence the binding of EGCG to the Omicron S protein RBD (Fig. 5). Consistent with the in vitro analysis, in silico molecular docking analysis showed that TFDG formed a stable association with the Omicron RBD of the S protein of the BA.2 lineage of Group 1’ with Y453, F486, and Q493R in Patch 1, Y449, Q498R, and N501Y in Patch 2. In contrast, the G446S substitution in BA.2.75 lineage of Group 2’ suggested that TFDG mainly interacted with Patch 2 (G446S, Y449, and Q498R in Patch 2). We speculate that the G446S substitution makes it difficult for TFDG to interact with patch 1 of the Omicron RBD of the S protein. On the other hand, on the XBB.1 lineage of Group 2’, the F490S substitution makes it possible to interact with patch 1 of the Omicron RBD of the S protein (Fig. 6e,f). Therefore, we calculated the binding free energy including contributions such as solvation, entropy, and enthalpy terms based on the polar interaction energy as the interaction of TFDG with each omicron RBD of the S protein. As a result, the interaction energy of the XBB.1 lineage (− 89.67 kcal/mol) is weaker than that of the BA.2 lineage (− 108.41 kcal/mol) and more substantial than that of the BA.2.75 lineage (− 89.13 kcal/mol).

These results explain the consistency between in silico and in vitro experiments concerning the TFDG's inhibition for the Omicron RBD of S protein binding with ACE2.

As Han et al.^25^ explained in the limitations of their study, we also focused on the interaction between the Omicron RBD of the S protein and hACE2 in our i*n silico* molecular docking analysis. We should consider that those mutations in other regions of the Omicron RBD of the S protein may also affect GCG, EGCG, and TFDG defense against viral infection.

Our clinical study showed that BA.1 virus was strongly inactivated in vitro by saliva from healthy volunteers who had just consumed a candy containing either green tea or black tea (Fig. 7). This is consistent with our previous study that catechins and catechin derivatives inactivate SARS-CoV-2 even in the presence of human saliva^18^. A candy containing green tea or black tea may be useful for inactivating virus if infected persons consume it, to decrease virus load in the oral cavity and gastrointestinal tract of the infected person, as well as to prevent spread of the virus from the infected persons to nearby noninfected persons. It has been reported that virus infect salivary grand and other oral tissues to propagate^6,7^. But the virus inactivating effect was not demonstrated in the saliva collected from healthy volunteers 5 or 15 min after the cessation of the candy consumption (Fig. 7). This may be due to high flow rates of saliva in heathy volunteers^31^.

These results indicate specific amino acid substitutions in Omicron RBDs that crucially influence the binding of EGCG/TFDG to the RBDs and different susceptibility of each Omicron subvariant to EGCG/TFDG. The study may suggest molecular basis for potential usefulness of tea catechins and their derivatives in suppression of transmission of mutant viruses that could emerge in the future and cause next pandemic.

## Materials and methods

### Virus, cells, and culture medium

The SARS-CoV-2 shown in Supplementary Table S2 were kindly provided from Japan National Institute of Infectious Diseases (Tokyo, Japan) and propagated using VeroE6/TMPRSS2 cells^32^ that were obtained from Japanese Collection of Research Bioresources Cell Bank, National Institute of Biomedical Innovation (Osaka, Japan). Mutations in the spike protein of each subvariant are shown in Supplementary Fig. S1. Cells were cultured in Dulbecco’s modified Eagle’s minimum essential medium (DMEM) (Nissui Pharmaceutical Co. Ltd., Tokyo, Japan) supplemented with G418 disulfate (1 mg/mL), penicillin (100 units/mL), streptomycin (100 μg/mL), 5% fetal bovine serum at 37 °C in a 5% CO_2_/95% humidified atmosphere.

### Tea and chemicals

Commercially available green tea leaves, powdered *Matcha* and black tea leaves were purchased from a local grocery store. To prepare “original tea beverage”, green tea was brewed by pouring 25 mL prewarmed hot water (70 °C) over 1 g green tea leaves for 2 min. *Matcha* green tea was prepared by whisking 1 g powdered *Matcha* in 30 mL hot water (80 °C). Black tea was brewed by pouring 300 mL boiled water (95–100 °C) over 10 g black tea leaves for 2 min. After the brewing or whisking, all tea samples were filtrated through 0.45 μm cellulose acetate filters and equilibrated to room temperature before use. Bottled green tea beverage was a commercial product by ITO EN ltd. and purchased from a grocery store (Supplementary Table S1). Chemical compounds were purchased from Nagara Science (Gifu, Japan) as shown in the Supplementary Table S3.

### TCID_50_ assay for virus treated with tea

The “original tea beverage” was placed into 96-well-plates at 180 μL/well (N = 3). SARS-CoV-2 suspension was added to the plate at 10^5^ TCID_50_/20 μL/well (vol:vol = 9:1). Thus, virus was treated with a final concentration of × 9/10 (90%) of the original tea beverage. After incubation for 10 s, the virus/tea mixtures were serially diluted tenfold with MS. Chilled on ice, 50 μL of each sample was added to the VeroE6/TMPRSS2 cells that had been seeded into 96-well-plates at 5 × 10^4^/100 μL/well a day before (N = 4). Cells were incubated for 1 h, followed by replacement of the supernatant by fresh 100 μL MS (DMEM supplemented with 0.5% FBS). After culture for 3 days, cytopathic effect (CPE) (cell death caused by Omicron variants of SARS-CoV-2) was observed under a phase contrast microscope^33^.

### Calculation of TCID_50_ values

TCID_50_ values were calculated by Reed–Muench method as described elsewhere^17^. If one or more triplicate wells of the lowest dilution of a sample did not show CPE, the highest possible average of TCID_50_ value was calculated for the sample.

### TCID_50_ assay for virus treated with chemical compounds

In some experiments, catechins and theaflavins were dissolved in MS, and virus suspension in DMEM (1.5 × 10^6^ TCID_50_/50 μL) was mixed with the catechin/theaflavin solutions for 10 s. The concentrations of catechins and theaflavins had been adjusted to the same concentrations in typical bottled green tea beverage and black tea, respectively (Supplementary Table S1). In other experiments, virus was treated with EGCG, GCG, and TFDG solutions at various concentrations for 10 s. Immediately after the treatment, the virus/chemical mixtures were serially diluted tenfold with MS, and TCID_50_ assay and calculation of TCID_50_ values were performed as above. In other experiments, “Pre” and “Mix” protocols were performed. In some groups (Pre), VeroE6/TMPRSS2 cells were pre-treated with various concentration of EGCG for 1 h, followed by washing and infection with non-treated Omicron BA.1 virus (10^5^TCID_50_/25 µL) for 1 h. In other groups (Mix), non-treated VeroE6/TMPRSS2 cells were infected for 1 h with the Omicron BA.1 virus (10^5^TCID_50_/25 µL) that had been pre-treated with the indicated concentration of EGCG for 10 s. In a control group (DW), non-treated VeroE6/TMPRSS2 cells were infected with DW-treated Omicron BA.1 virus (10^5^TCID_50_/25 µL) for 1 h. After washing, cells were cultured for 3 days, and virus titer was determined as above.

### Neutralizing assay

Neutralizing assay was performed using SARS-CoV-2 Surrogate Virus Neutralization Test Kit (GenScript, Piscataway, NJ, USA) by “mix protocol” and “pretreatment protocol”. In the “mix protocol”, various concentrations of EGCG, GCG or TFDG, or distilled water as a control, were mixed with a horseradish peroxidase (HRP)-conjugated recombinant BA.1 RBD fragment at a volume ratio of 1:1 at 37 °C for 1 min. One hundred µL of each mixture was added to the wells pre-coated with human ACE2 protein. In the “pretreatment protocol”, ACE2-pre-coated wells were treated with various concentrations of EGCG, or distilled water as a control, for 30 min, followed by washing and an addition of HRP- BA.1 RBD to the wells. After incubation at 37 °C for 15 min, wells were washed and 100 μL of 3, 3′, 5, 5′-tetramethyl-benzidene (TMB) solution was added. Following incubation in the dark at 20–25 °C for 15 min, absorbance at 450 nm was measured.

### In silico analysis

The construction of the three-dimensional structure of the hACE2 interaction site of the surface spike glycoprotein (S-protein) of SARS-CoV-2 and the extracellular peptidase domain of ACE2 complex was performed with MOE, version 2022.02 (CCG Inc, Montreal, Canada) based on the Brookhaven Protein Databank 7WBP. Molecular mechanics calculations for this complex were performed to obtain the local minimum structure of the RBD of S-proteins of BA.1, BA.2, and BA.2.75 lineages for molecular docking simulations with EGCG (PubChem CID: 65,064), and TFDG (PubChem CID: 136,277,567) using the Amber99 force field in MOE.

### Study with human materials

Clinical studies were approved by ethical committee of the ITO EN, ltd. All experiments were performed in accordance with relevant named guidelines and regulations. Informed consent was obtained from all volunteers. Candies were originally produced, and the contents are shown in the Supplementary Table S4). Seven healthy volunteers (ages, 38.1 ± 3.6 (AV ± SE, n = 7), gender F/M = 3/4) were starved for 2 h and consumed a placebo candy (without tea) or a candy containing either green tea or black tea for 5 min. Saliva was collected from the volunteers immediately before the candy consumption, and 0, 5 or 15 min after cessation of the candy consumption. The samples were vortexed and centrifuged at × 20,000 *g* for 5 min at 4 °C to collect supernatant. Virus suspension of Omicron BA.1 (1.5 × 10^5^ TCID_50_/25 μL) was mixed with 225 μL of saliva or water for 10 s. Immediately, the mixtures were serially diluted at tenfold with MS in quadruplicate in 96-well-plates. Chilled on ice, each sample was added to the VeroE6/TMPRSS2 cells and TCID_50_ assay and TCID_50_ evaluation were performed as above.

### Statistical analysis

Statistical significance was analyzed by Tukey’s multiple comparison and ANOVA (analysis of variance) using GraphPad Prism 9 manufactured by GraphPad Software. *p* < 0.05 was considered significant.

### Informed consent statement

Clinical studies were approved by the ethical committee of the ITO EN, ltd.

### Supplementary Information

Supplementary Information.

## Data Availability

The datasets used and/or analyzed during the current study are available from the corresponding author on reasonable request.
