# Supplementary material for: Effects of tea, catechins and catechin derivatives on Omicron subvariants of SARS-CoV-2
Source: Sci Rep. 2023 Oct 3;13:16577. doi: 10.1038/s41598-023-43563-3 (PMC10547759; doi:10.1038/s41598-023-43563-3)
Supplement: Supplementary file 1 — Supplementary Information. [file 41598_2023_43563_MOESM1_ESM.pdf]

	Catechins								Theaflavins			
	C	EC	CG	ECG	GC	EGC	GCG	EGCG	TF	TF3G	TF3'G	TFDG
Bottled green tea beverage	66	41	183	199	232	102	753	641	-	-	-	-
Black tea	-	-	-	-	-	-	-	-	60.4	76.6	35.8	58.7

Supplementary Table S1
Concentrations (μM) of catechins and theaflavins in bottled green tea beverage and black tea.
"-", not determined.

Pango lineage	Strain name	GISAID ID
BA.1.18	hCoV-19/Japan/TY38-873/2021	EPI_ISL_7418017
BA.2	hCoV-19/Japan/TY40-385/2022	EPI_ISL_9595859
XE	hCoV-19/Japan/TY41-686/2022	EPI_ISL_12703378
BA.5	hCoV-19/Japan/TY41-702/2022	EPI_ISL_13241867
BA.2.75	hCoV-19/Japan/TY41-716/2022	EPI_ISL_13969765
XBB.1	hCoV-19/Japan/TY41-795-P1/2022	EPI_ISL_15669344
BQ.1.1	hCoV-19/Japan/TY41-796-P1/2022	EPI_ISL_15579783

Supplementary Table S2
Virus strains used in this study. All viruses were kindly provided by Japan National Institute of Infectious Diseases (Tokyo, Japan).

	Producer	Cat Number
C	Nagara Science	NH021102
EC	Nagara Science	NH020102
CG	Nagara Science	NH021302
ECG	Nagara Science	NH020302
GC	Nagara Science	NH021202
EGC	Nagara Science	NH020202
GCG	Nagara Science	NH021402
EGCG	Nagara Science	NH020602
TF	Nagara Science	NH030101
TF3G	Nagara Science	NH030201
TF3’G	Nagara Science	NH030301
TFDG	Nagara Science	NH030401

Supplementary Table S3
Chemical compounds used in the study.

	Ingredients	Components in a piece of candy
Placebo candy (3.5 g)	50 % white granulated sugar, and 50 % starch syrup	Energy, 13.7 kcal<br>Protein, 0.0 g<br>Lipids, 0.0 g<br>Carbohydrates, 3.2 g<br>Sodium chloride equivalent, 0.0 g
Green tea candy (3.5 g)	47.5 % white granulated sugar, 47.5 % starch syrup, and 5% green tea extract	Energy, 12.9 kcal<br>Protein, 0.0 g<br>Lipids, 0.0 g<br>Carbohydrates, 3.2 g<br>Sodium chloride equivalent, 0.0 g<br>Catechins, 140 mg
Black tea candy (3.5 g)	47.5 % white granulated sugar, 47.5 % starch syrup, and 5% black tea extract	Energy, 13.0 kcal<br>Protein, 0.0 g<br>Lipids, 0.0 g<br>Carbohydrates, 3.2 g<br>Sodium chloride equivalent, 0.0 g<br>Theaflavins, 50 mg

Supplementary Table S4
Contents of the candies used in this study.

Concentrations (µM) of catechins in the saliva from volunteers who consumed a candy containing green tea

	GC	EGC	C	EC	EGCG	GCG	ECG	CG	CAF
Average	159.2	1462.4	73.8	616.5	5,365.8	293.4	1,672.9	54.7	206.8
SD	72.3	627.7	50.6	195.4	6,374.4	347.0	2,041.7	67.9	137.4

Concentrations (µM) of catechins and theaflavins in the saliva from volunteers who consumed a candy containing black tea

	GC	EGC	C	EC	EGCG	GCG	ECG	CG	CAF	TF	TF3G	TF3'G	TFDG
Average	18.7	225.1	35.1	345.7	566.9	53.4	721.2	18.8	65.1	329.8	567.5	144.6	501.9
SD	10.0	122.3	14.0	141.3	441.5	38.2	539.2	13.3	21.9	133.3	263.9	65.0	231.1

Supplementary Table S5
Concentrations of catechins and theaflavins in the saliva from volunteers collected 0 min after cessation of the green tea candy or black tea candy consumption.

Supplementary Figure S1

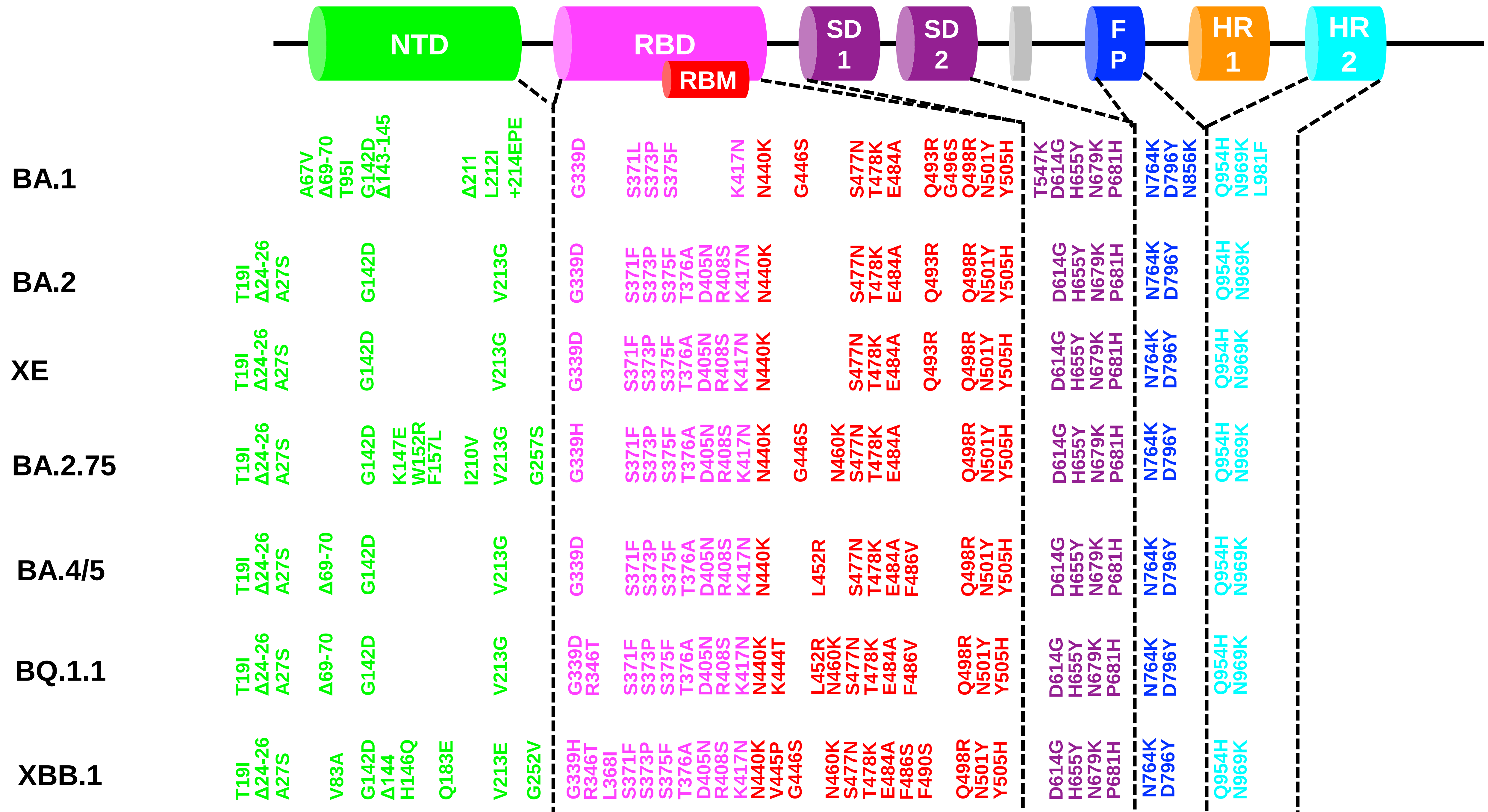

Legend to the Supplementary Figure S1
Mutations in RBDs in omicron subvariants are shown.

Supplementary Figure S2

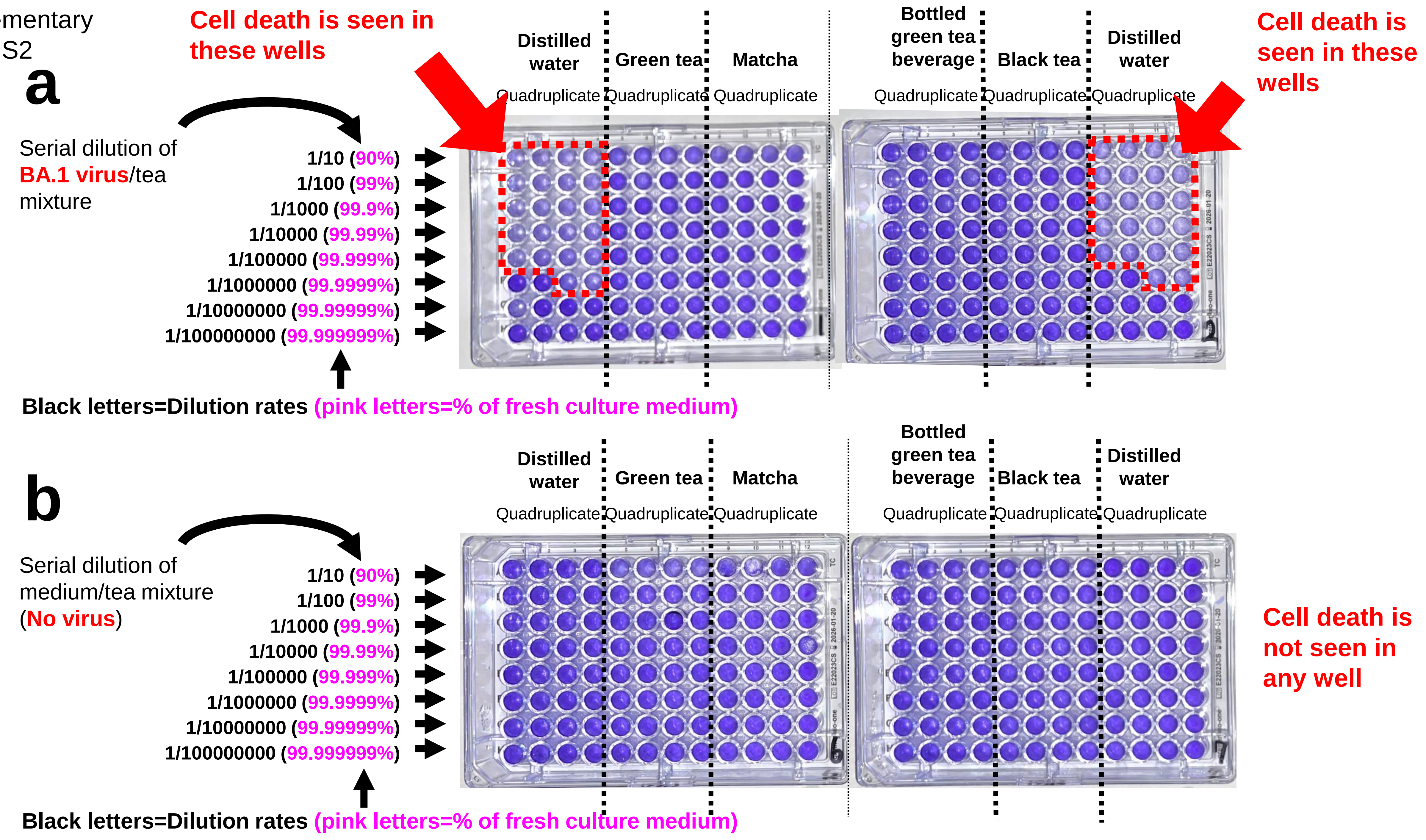

Legend to the Supplementary Figure S2.

(a) Raw data of an experiment that was performed exactly the same as Fig. 1a. (b) Raw data of an experiment that was performed exactly the same as in (a) but the only difference was that virus was not added.

In (a), the cells in the well surrounded by the red lines (distilled water (DW)-treated virus-infected cells in upper four rows) were dead. In contrast, cells in any wells in (b) were not killed, strongly suggesting that the dead cells in (a) were killed by DW-treated virus. In sharp contrast, virus treated with green tea, Matcha, or black tea didn’t kill the cells in any well (even in upper four rows) in (a). Thus, consistent with Fig. 1a, treatment with tea reduced virus titer to $1/10^4$ or lesser compared with the treatment with DW. The results of (b) also demonstrate that potential toxicities of tea, even if they exist, did not damage cells to influence the results of TCID50 assay under these experimental conditions.

Supplementary Figure S3

(+)-Catechin
(C)

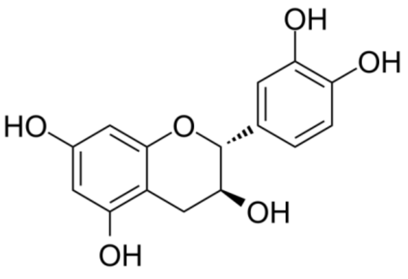

(-)-Epicatechin
(EC)

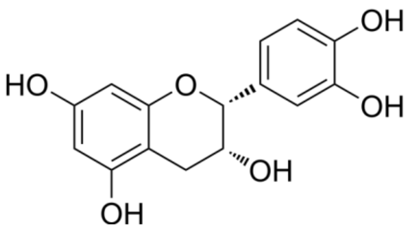

(-)-Catechin gallate
(CG)

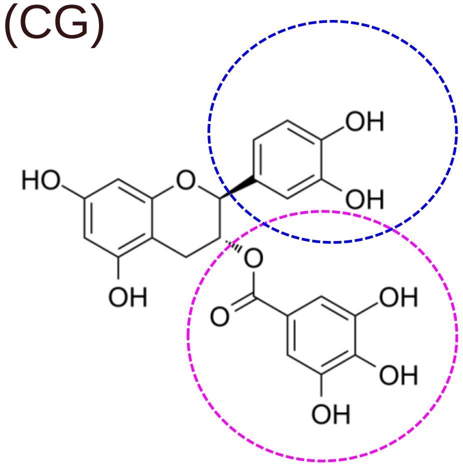

(-)-Epicatechin gallate
(ECG)

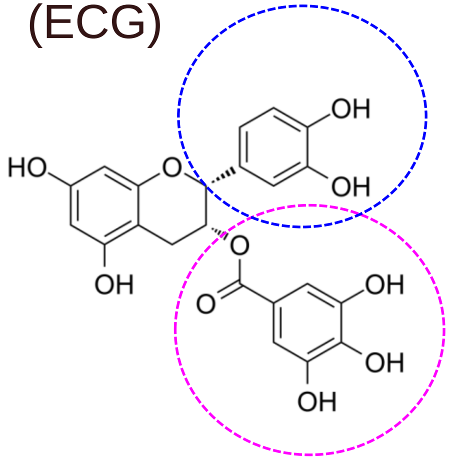

(-)-Gallocatechin
(GC)

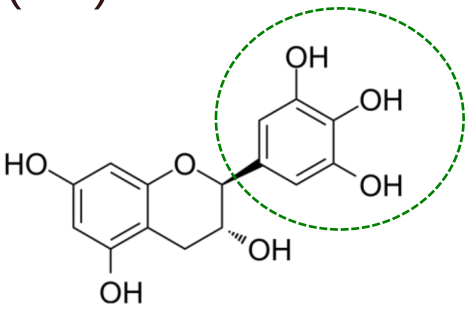

(-)-Epigallocatechin
(EGC)

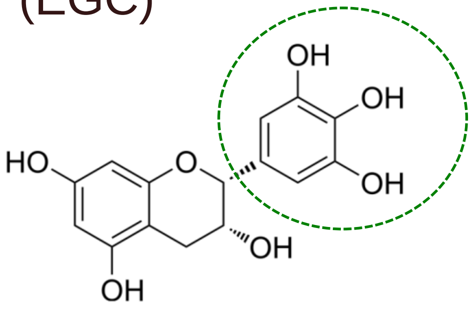

(-)-Gallocatechin
gallate (GCG)

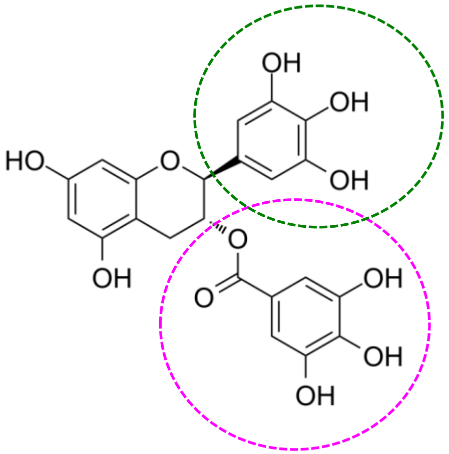

(-)-Epigallocatechin
gallate (EGCG)

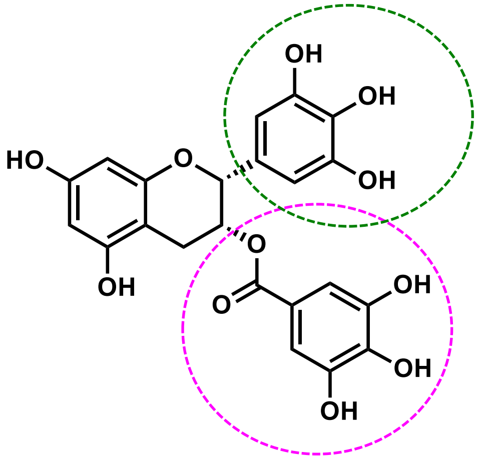

Pink circles: galloyl groups
Green circles: 3,4,5-trihydroxyphenyl groups
Blue circles: 3,4-dihydroxyphenyl groups

Legend to the Supplementary Figure S3
Chemical formulae of tea catechins are shown.

Supplementary Figure S4

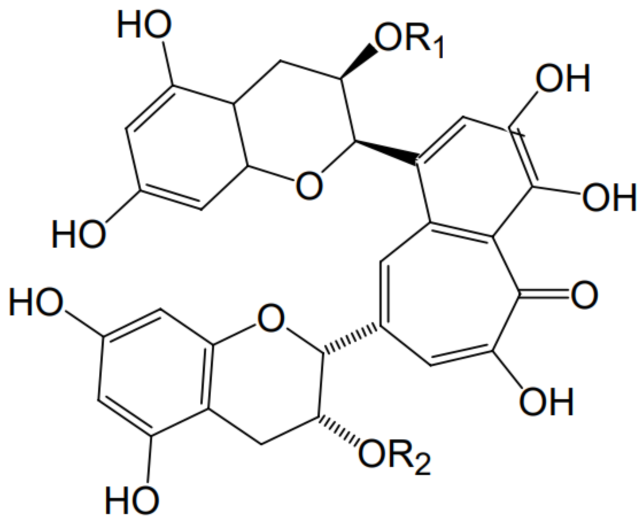

Theaflavins

Theaflavins	R_1	R_2
Theaflavin	H	H
Theaflavin-3-gallate	G	H
Theaflavin-3'-gallate	H	G
Theaflavin-3,3'-digallate	G	G

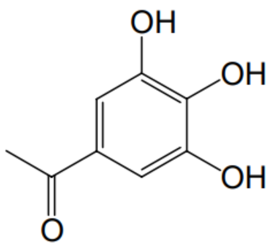

Galloyl (G)

Legend to the Supplementary Figure S4
Chemical formulae of theaflavins are shown.

Supplementary Fig. S5

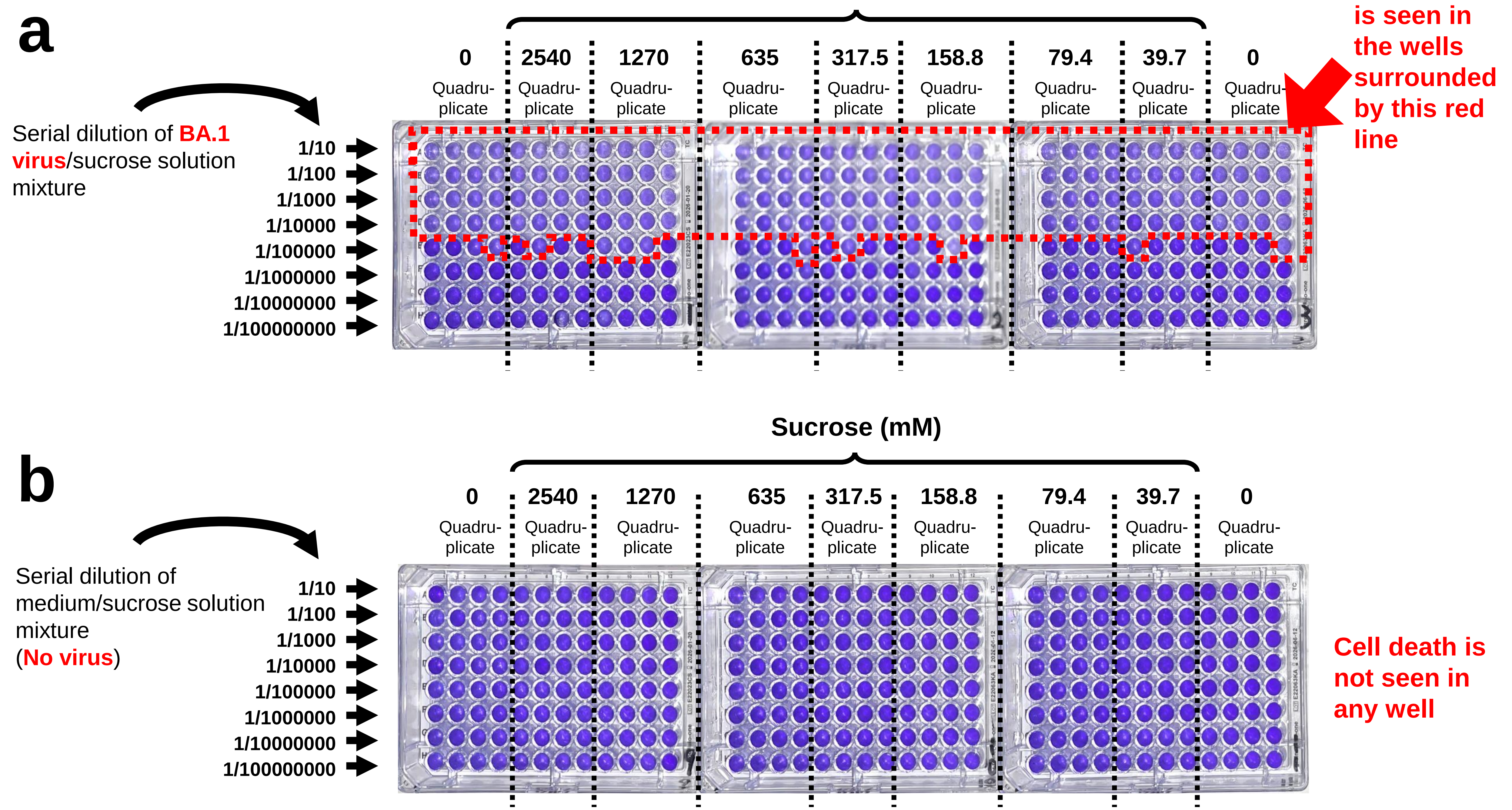

Legend to the Supplementary Fig. S5.
(a) Raw data of the TCID50 assay that was performed exactly the same as in Fig. 7, except that human saliva was replaced by various concentrations of sucrose solutions (0 to 2,540 mM) (2,540 mM is almost the same as the saturation concentration of sucrose at room temperature). (b) Raw data of an experiment that was performed exactly the same as (a) but the only difference was that virus was not added.

In (a), cells in the wells in upper four or five rows (surrounded by the red line) were dead regardless of the concentration of the sucrose (0 to 2,540 mM) used to treat virus. The results show that the virus titers of every groups were almost the same (approximately 5 x 10^4 $TCID_{50}$/50 µL) independently of the sucrose concentrations. Thus, sucrose didn’t inactivate the virus, nor act on cells to induce anti-virus effects. Sucrose didn’t promote virus infection, either.
In (b), cell death was not seen in any well, indicating that the cell death in (a) was not induced by sucrose but by virus. Also, it was demonstrated that the sucrose didn’t damage the cells to influence virus titer estimation in this experimental setting.
Collectively, sucrose does not influence virus titer determination by $TCID_{50}$ assay by inactivating the virus, damaging cells, or rendering the cells resistant to the virus, even at the highest concentration of sucrose.
